# Improvement Effect of Water-Based Organic Polymer on the Strength Properties of Fiber Glass Reinforced Sand

**DOI:** 10.3390/polym10080836

**Published:** 2018-07-30

**Authors:** Jin Liu, Zezhuo Song, Yi Lu, Qiongya Wang, Fanxuan Kong, Fan Bu, Debi Prasanna Kanungo, Shaorui Sun

**Affiliations:** 1 School of Earth Sciences and Engineering, Hohai University, Nanjing 210098, China; szzhhu@163.com (Z.S.); hhu_wqy@163.com (Q.W.); kfxhhu@163.com (F.K.); bf_hhu@163.com (F.B.); sunsrhhu@163.com (S.S.); 2 Key Laboratory of Earth Fissures Geological Disaster, Ministry of Land and Resource (Geological Survey of Jiangsu Province), Nanjing 210049, China; lynju@163.com; 3 CSIR-Central Building Research Institute (CBRI), Roorkee 247667, India; debi.kanungo@gmail.com

**Keywords:** water-based organic polymer, fiber glass, sand, strength property, reinforcement mechanism

## Abstract

The mechanical properties of sandy soil can be effectively improved by the incorporation of water-based polymer and glass fibers. In order to study the reinforcement effects of a type of water-based organic polymer and fiber glass on sand, three strength tests (unconfined compression test, direct shear test and tensile test) and scanning electron microscopy were carried out. A series of polymer content, fiber content and dry density were selected for the tests. The results revealed that the composite reinforcement of water-based organic polymer and fiber glass can improve the strength. With an increase in polymer content and fiber content, the unconfined compression strength, the cohesion, and the tensile strength increase. The internal friction angles maintain a relatively stable state. All three strength properties increase with an increase in dry density. The results can be considered as the reference for sand reinforced engineering.

## 1. Introduction

Natural sand material has certain deficiencies in strength, thereby affecting the stability of foundations and slopes. The research and application of new materials that can improve the strength of sand has become a hot topic in the world. At present, the materials used to reinforce sand are mainly fiber, geosynthetics [1,2], geotextiles [3,4], plant [5,6] and so on. Among them, fiber is noted for its tensile strength and acid- and alkaline-resistance. In recent years, more research about fiber as a physical reinforcing material has been carried out [7,8,9,10]. The fibers added into the sand can be divided into natural fibers (including straw fiber, cotton fiber and wool fiber) and artificial synthetic fibers. In the natural environment, the hydrolysis of natural fiber is very common, which reduces their strength and affects the reinforcing effect. Among the artificial synthetic fibers, fiber glass is one of the most commonly used fibers in engineering practices. The mechanical properties, such as the tensile strength of fiber glass, is significantly higher than that of other artificial synthetic fibers. However, when fiber glass is used with loose sand, the integrity of fiber reinforced sand is not improved.

In recent years, some chemical synthetic materials have been used in sand reinforcement. The main chemicals used for sand reinforcement include lime [11,12], biological enzymes [13,14,15], some ions [16,17], and organic polymers. As a new type of sand reinforcement material, the organic polymer has the advantages of less incorporation, convenient transportation, simple construction, stable effect, and ecological and environmental protection. It has attracted the attention of many scholars and there are a number of important results published. Rezaeimalek et al. [18] studied the effect of a moisture-activated liquid polymer from the generic family of methylene diphenyl diisocyanates (MDI) on the strength of sandy soil through laboratory experiments. Hong and Lee [19] studied a kind of environment-friendly polymer called potassium polyacrylate to promote the growth of coastal vegetation, thereby reducing the cost of coastal erosion protection. Collins et al. [20] used polymer emulsions and fibrillated geo-fibers to study the erosion characteristics of sandy soils and analyzed their curing mechanisms.

In this paper, a type of water-based organic polymer and fiber glass was used as the sand reinforcement material. Three strength tests (unconfined compression test, direct shear test and tensile test) and scanning electron microscopy were applied to identify the reinforcement effects. A series of polymer content, fiber content and dry density were selected for the tests. Finally, the reinforcement mechanism was analyzed. The research results can be considered as the reference for sand reinforced engineering.

## 2. Materials

### 2.1. Sand

**Figure 1:**
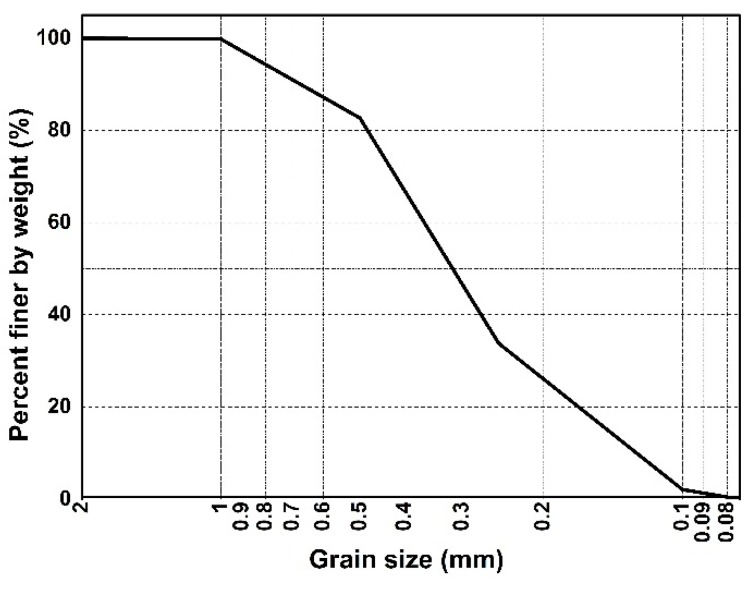
The particle size distribution curve of sand.

The sand used in this study was taken from Nanjing city of Jiangsu province, China. The basic physical parameters were determined according to the American Society of Testing Materials (ASTM) standards in the laboratory. According to the test results, the particle size distribution curve of sand is shown in Figure 1. The specific gravity (*G*s), maximum dry density (*ρ*_max_), minimum dry density (*ρ*_min_), maximum void ratio (*e*_max_), minimum void ratio (*e*_min_), mean grain size (*D*_50_), compression exponent (*C*_c_), and uniformity coefficient (*C*_u_) are shown in Table 1.

### 2.2. Water-Based Organic Polymer

**Figure 2:**
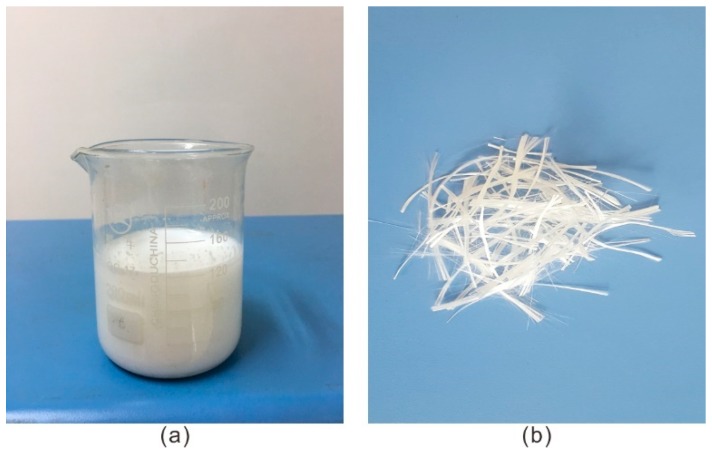
The material: (**a**) Water-based organic polymer; (**b**) Fiber glass.

A type of water-based organic polymer (Figure 2a) was prepared for this study. Its main constituent is polyurethane resin, and it also contains an enormous amount of the functional group –NCO. It is a light-yellow colored oil liquid with a pH of 6–7, viscosity of 650–700 MPa·s, specific gravity of 1.18 g/cm^3^, solid content of 85%, coagulation time of 30–1800 s and water absorption of 40 times. The coagulation time decreases with an increase in the concentration. It is an environmentally friendly product with no additional pollutants. The proportion of the polymer weight to the weight of the dry sand (*P*_s_) is given as:

(1) $$P_{s}=\frac{W_{p}}{W_{S}}\times 100\%,$$

where, *w*_p_ (g) is the polymer weight and *w*_s_ (g) is the dry sand weight.

The synthesis of the water-based organic polymer is as follows. Polyoxypropylene glycol (PPG) with number-average molecular weight of approximately 3000, polyoxyethylene glycol (PEG) with number-average molecular weight of approximately 2000, polylactic acid diol with number-average molecular weight of approximately 1000 and toluene were added to the reaction still. These materials were under air distillation at 135 °C until the toluene was close to steaming. Then, the vacuum distillation was adopted to eliminate residual water and toluene in the reaction system. The entire reaction system was then cooled to room temperature and a reflow condenser was installed. N_2_ was injected into the reaction system and oil sealing was carried out. Toluene diisocyanate was added to the reaction system and stirred at 95 °C for 2.5 h. Subsequently, the reaction system was cooled down to room temperature. Ethyl acetate was added to the reaction system and stirred at room temperature for 2 h. Sodium dodecyl sulfate was added as a surfactant before the final product was obtained. The water-based organic polymer used in this paper was obtained after sufficient stirring.

### 2.3. Fiber Glass

The fiber glass (Yongxing Fiber Glass company, Nanjing, China) used in this study can be seen in Figure 2b, and the length of the fiber glass is 18 mm. Before being dispersed into specimens, the strip fiber glass was torn and then evenly incorporated into the sand. The fiber glass mixed with the sand was filamentous. The basic physical and mechanical parameters of the fiber glass, which were determined and provided by the manufacturer, are shown in Table 2.

## 3. Experimental Methods

The sand was air-dried, crushed, and sieved at 2 mm in the laboratory. Subsequently, a certain amount of sand, fiber glass, water and water-based organic polymer were mixed to make the specimens that had a certain shape. A water content of 10% was used for the mixing process of all specimens. The specimens were kept in the curing box with a temperature of 20 °C and constant humidity for 48 h. The strength tests, which include an unconfined compression test, direct shear test and tensile test, were carried out after curing [21]. All the tests were carried out in an environment of about 50% humidity. The relevant parameters of the specimens are given in Table 3. For the specimens at each relevant parameter, all the tests were repeated at least five times.

### 3.1. Unconfined Compression Test

**Figure 3:**
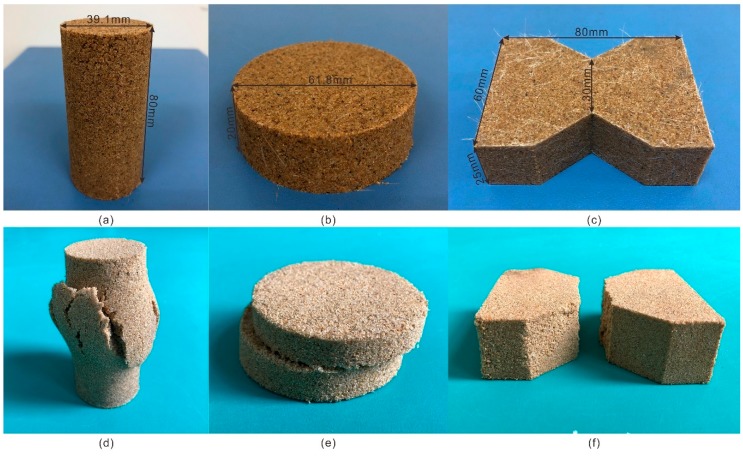
Photographs of compacted and broken specimens: (**a**) Compacted unconfined compression test specimen; (**b**) Compacted direct shear test specimen; (**c**) Compacted tensile test specimen; (**d**) Broken unconfined compression test specimen; (**e**) Broken direct shear test specimen; (**f**) Broken tensile test specimen.

The specimens (Figure 3a) involved in the unconfined compression test (S1–S20) were prepared with the static compaction method based on ASTM standards (ASTMD2166-00). In the test, the YYW-2 unconfined compressional strain control device (Nanjing Nantu Instrument Equipment Co. LTD, Nanjing, China) was used to obtain the experimental data. The controlling strain rate was 2.4 mm/min and the test for one specimen lasted for about 7 min. After the test, the specimens suffered axial failure (Figure 3d). In this study, the residual strength refers to the minimum strength value after sample failure. The elasticity modulus is the ratio of axial stress, which is half of the unconfined compression strength, to its corresponding axial strain.

### 3.2. Direct Shear Test

The specimens (Figure 3b) involved in the direct shear test (S1–S20) were prepared with the static compaction method based on ASTM standards (ASTM D3080-98). In the test, the ZJ-type strain controlled direct shear apparatus (Nanjing Nantu Instrument Equipment Co. LTD, Nanjing, China) was used to obtain the experimental data. The lower half of the shear box was kept fixed during the experiment and the upper half was subjected to horizontal displacement under the fixed rate of 2.4 mm/min. The maximum horizontal displacement was 14 mm in the test. After the test, the specimens suffered shear failure (Figure 3e). The shear strength parameters (cohesion and internal friction angle) were calculated by the experimental data based on Coulomb’s law. Four normal pressures, which included 100, 200, 300 and 400 kPa in this study, and four corresponding peak shear stresses were used to fit a straight line. The intercept and slope of the straight line are the cohesion and the tangent value of the internal friction angle, respectively. For example, the corresponding peak shear stress of a group of specimens at the normal pressure of 100, 200, 300 and 400 kPa are 100, 150, 200, and 250 kPa, respectively. The linear equation obtained by linear fitting with these data is *y* = 50 + 0.5*x*. So, the cohesion is 50 kPa and the internal friction angle is 26.57°.

### 3.3. Tensile Test

The specimens of the tensile test (S1–S20) are 8-sided, with 80 mm long and 25 mm high sides (Figure 3c). A neck at the center of the specimens was formed to reduce the width from 60 to 30 mm, which allow the failure to take place at the center during the test. The specimens were prepared by the customized mold. The mold is also a part of the tensile test apparatus [22]. It was used to connect the sample to the tensile test apparatus as a whole. The tensile force was recorded by the force gauge during the whole test. After the test, the specimens suffered failure (Figure 3f). The tensile strength was calculated using the following equation:

(2) $$\sigma_{t}=\frac{2T+W_{1}+W_{2}}{2S},$$

where, *σ*_t_ (kPa) is the tensile strength, *T* (N) is the maximum tensile force, *W*_1_ (N) is the weight of specimen, *W*_2_ (N) is the weight of the mold and *S* (m^2^) is the cross-sectional area.

## 4. Results and Analysis

### 4.1. The Results of the Strength Tests

The strength of sand reinforced by the water-based organic polymer and fiber glass was thoroughly studied in the tests. According to the experimental methods, the experimental research on the strength of reinforced sand under different fiber content, different polymer content and different dry density was carried out. The results of these tests are shown in Table 4.

### 4.2. Unconfined Compression Test

#### 4.2.1. Effect of Polymer Content

**Figure 4:**
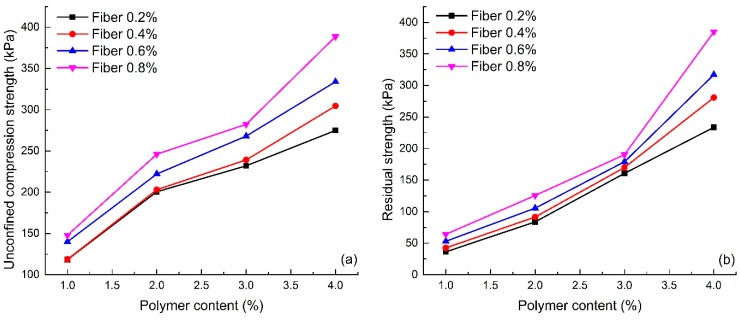
Effect of polymer content on unconfined compression test: (**a**) Unconfined compression strength; (**b**) Residual strength.

Figure 4 shows the effect of polymer content on the sand reinforced by the water-based organic polymer and fiber glass (S1–S16). The figure shows that when the fiber content remains same, the unconfined compression strength and the residual strength variations are similar, and the trend is observed to be increasing with an increase in polymer content. For instance, the unconfined compression strength of specimens with 0.4% fiber content increases from 118.57 to 304.61 kPa, when the polymer content increases from 1% to 4%. The growth rate is about 159.90%. It indicates that the water-based organic polymer contributed to the significant improvement of the unconfined compression strength of the sand.

**Figure 5:**
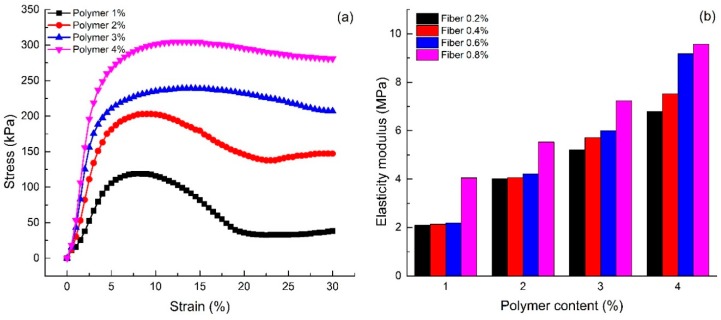
Effect of polymer content on unconfined compression test: (**a**) Stress-strain curves; (**b**) Elasticity modulus.

Figure 5a is the stress-strain curves with different polymer content and 0.4% fiber content (S5–S8). The figure shows that the stress-strain curves of the sand specimens are approximately linear at the initial stage, and the axial stress increases with an increase in axial strain at different rates. The higher the polymer content, the higher the rate. After the sample was broken, all the stress-strain curves have a downward trend. The curve of specimens with high polymer content is more gentle than that of specimens with lower polymer content.

Figure 5b illustrates the elasticity modulus of the specimens (S1–S16) in the unconfined compression test. As seen, the elasticity modulus of each fiber content increased with increasing polymer content. The elasticity modulus of 4% polymer content specimens with four fiber contents 0.2%, 0.4%, 0.6% and 0.8% are 6.79, 7.52, 9.18 and 9.56 MPa, respectively. They are 3.24, 3.53, 4.20 and 2.36 times higher compared to the specimens at 1% polymer content. This indicates that the ability to resist the elastic deformation of reinforced sand is strengthened with an increase in polymer content.

#### 4.2.2. Effect of Fiber Content

**Figure 6:**
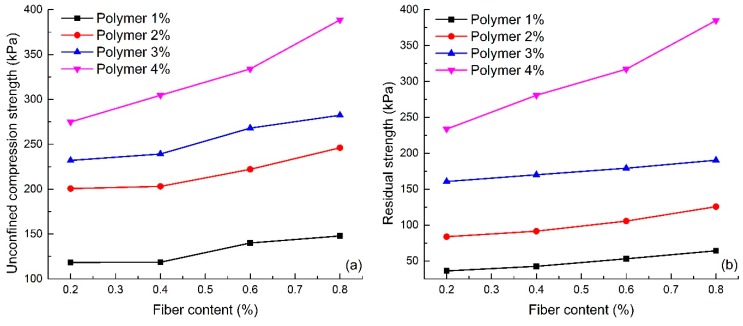
Effect of fiber content on unconfined compression test: (**a**) Unconfined compression strength; (**b**) Residual strength.

Figure 6 shows the effect of fiber content on the unconfined compression strength and the residual strength of reinforced sand (S1–S16). The figure shows that with the same polymer content, both the unconfined compression strength and the residual strength of the reinforced sand increases with an increase in fiber content. The values of unconfined compression strength and residual strength for specimens with 2% polymer content increase from 200.60 to 246.05 kPa and from 83.79 to 125.60 kPa respectively. This increase in unconfined compression strength and residual strength demonstrates the effectiveness of fiber glass in the sand reinforcement.

**Figure 7:**
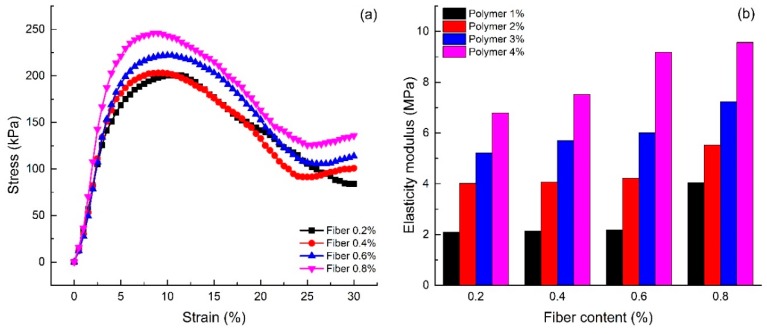
Effect of fiber content on unconfined compression test: (**a**) Stress-strain curves; (**b**) Elasticity modulus.

Figure 7a shows the stress-strain curves with 2% polymer content and different fiber contents (S2, S6, S10 and S14). Figure 7a shows that an increase in fiber content enhanced the unconfined compression strength of reinforced sand, but the extent of the increase in residual strength with an increase in fiber content was not significant. It can also be seen that the specimen with high fiber content exhibits more ductile behavior and has a smaller loss of post-peak strength than the specimen with low fiber content. In addition, Figure 7a shows that the initial stiffness of sand appears not to be affected by the addition of fiber. Figure 7b shows the elastic modulus of the reinforced sand with different fiber content (S1–S16). The figure shows that the elastic modulus increases with increasing fiber content, which also indicates that increasing the fiber content can improve the ability of sand to resist elastic deformation.

#### 4.2.3. Effect of Dry Density

**Figure 8:**
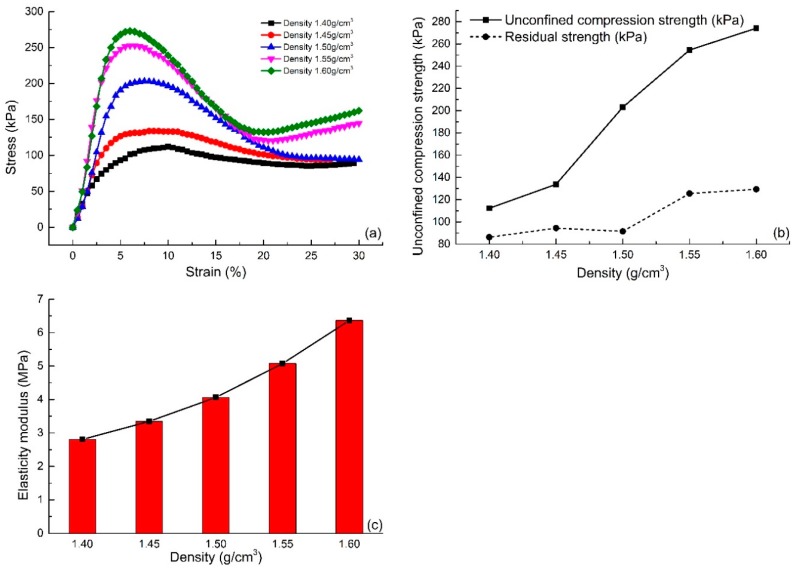
Effect of dry density on the unconfined compression test: (**a**) The stress-strain curves of different dry density; (**b**) The strength curves of different dry density; (**c**) The elasticity modulus curves of different dry density.

Figure 8a shows the stress-strain curve of the unconfined compression test of specimens with different dry densities (S6 and S17–S20). Figure 8a shows that the stress-strain curve of reinforced sand with high dry density is placed above that of reinforced sand with low dry density. The early stiffness is approximately equivalent. After the destruction of the specimen, the downward trend of the stress-strain curve of reinforced sand with high dry density is significantly more pronounced than that seen for the reinforced sand with low dry density. As shown in Figure 8a, the unconfined compression strength increases with an increase in dry density. It also shows that the reinforced sand with high dry density has obvious brittleness.

Figure 8b shows the strength curves of different dry densities. The figure shows that the unconfined compression strength and the residual strength of reinforced sand both increase with an increase in dry density, but with different trends. With the increase in dry density, the unconfined compressive strength of reinforced sand increases significantly. The growth rate is about 126.69%. However, the residual strength of reinforced sand grows slowly. The increase in the dry density of reinforced sand decreases the porosity between sand particles. Thus, the integrity of reinforced sand is improved and therein forces the sand to withstand greater pressure. The improvement in integrity also leads to the damaged form of reinforced sand as a result of brittle failure. Hence, the residual strength of reinforced sand could not be greatly improved. This can also be seen from the elastic modulus of reinforced sand with different dry densities. Figure 8c illustrates the elasticity modulus of the specimens with different dry densities. As shown, the elasticity modulus of 1.60 g/cm^3^ dry density is 6.36 MPa, which is 2.27 times compared to the reinforced sand at 1.40 g/cm^3^ dry density. With the increase in dry density, the polymer can enwrap the sand easily, which improves the elasticity of the sand.

### 4.3. Direct Shear Test

#### 4.3.1. Effect of Polymer Content

**Figure 9:**
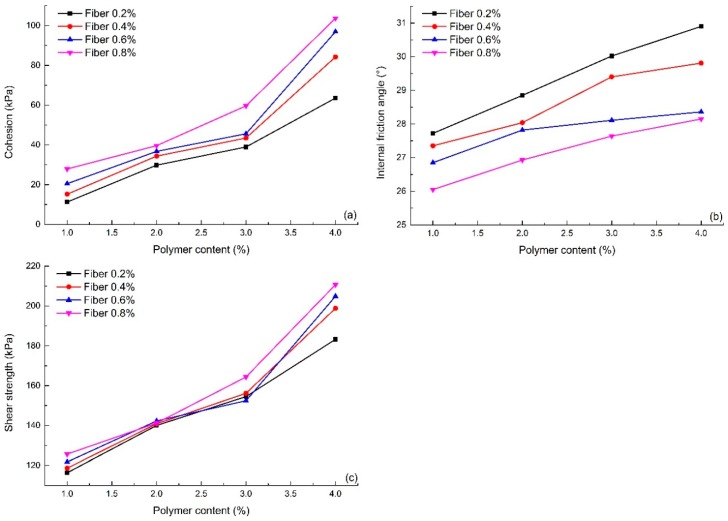
The variation curve with the change of polymer content: (**a**) Cohesion; (**b**) Internal friction angle; (**c**) Shear strength.

In order to analyze the effect of polymer content on shear strength parameters, the experimental results of S1–S16 were selected as representative, and were shown in Figure 9a,b.

It can be seen from Figure 9a that the cohesion of reinforced sand increases with an increase in polymer content, but the contribution of polymer content to the cohesion value in different intervals is obviously different. When the polymer content increased from 1% to 2%, the average cohesion value increased by 103.0%. While the polymer content increased from 2% to 3%, the cohesion value increased by 33.0%. The average growth rate of cohesion has declined significantly. When the polymer content increased from 3% to 4%, the average growth rate of cohesion is approximately 85.9%. It can be seen that the contribution of polymer to strength is not linear in relation to polymer content. Figure 9b shows that the change in polymer content has no obvious effect on the internal friction angle. Generally speaking, the internal friction angle of reinforced sand with high polymer content is slightly higher than that with low polymer content.

The shear strength can be calculated by the following equation:

(3) τ=σ·tanφ+c,

where, *τ* (kPa) is the shear strength of the reinforced sand, *σ* (kPa) is the vertical pressure (which includes 100, 200, 300 and 400 kPa in this study), φ (°) is the internal friction angle and *c* (kPa) is the cohesion.

The shear strength of reinforced sand under 200 kPa vertical pressure was calculated and the changing curve is shown in Figure 9c. Although the internal friction angle of the reinforced sand varies little with increasing polymer content, the shear strength still shows a significant increase. As seen, the shear strength of 4% polymer content specimens with four fiber contents 0.2%, 0.4%, 0.6% and 0.8% are 183.25, 198.81, 204.88 and 210.67 kPa, respectively. They are 1.57, 1.68, 1.68 and 1.68 times compared with the specimens at 1% polymer content.

#### 4.3.2. Effect of Fiber Content

**Figure 10:**
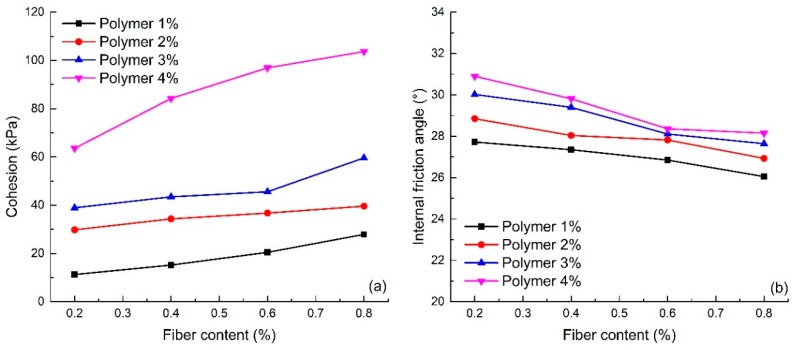
The variation curve of the shear strength parameters with the change of fiber content: (**a**) Cohesion; (**b**) Internal friction angle.

Figure 10 shows the relationship between shear strength parameters and fiber content (S1–S16). It shows that the fiber content plays an important role in the development of the shear strength parameters. The cohesion of reinforced sand increases with increasing fiber content (Figure 10a). When the polymer content is not greater than 3%, the cohesion growth trend is not obvious. However, with 4% polymer content, the cohesion increases faster with an increase in fiber content.

The change in the internal friction angle shows an opposite trend. With an increase of fiber content, the internal friction angle of reinforced sand maintains relative stability (Figure 10b). In general, it has a certain tendency to decrease, but the change in trend is small. This may be related to local fibers forming and overlapping with each other, resulting in a reduction of the interfacial friction coefficient.

#### 4.3.3. Effect of Dry Density

**Figure 11:**
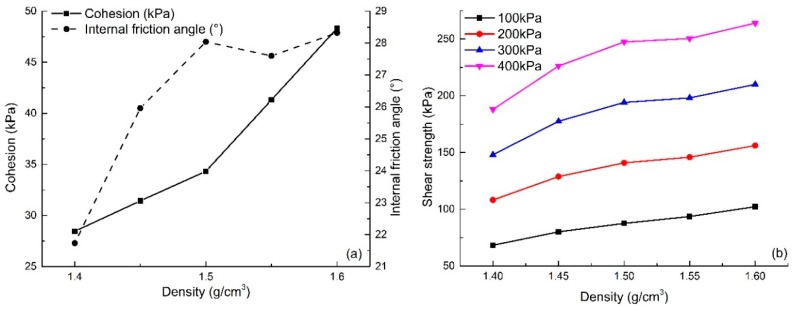
The variation curves with the change of dry density: (**a**) Cohesion and internal friction angle; (**b**) Shear strength.

In order to analyze the effect of dry density on shear strength parameters, the experimental results of reinforced sand with different dry densities have been selected and shown in Figure 11a (S6, S17–S20). The figure shows that an increase in dry density can effectively improve the cohesion and internal friction angle of reinforced sand when other conditions remain equal. To be specific, when the dry density increased from 1.4 to 1.6 g/cm^3^, the value of cohesion and internal friction angle increased by 69.9% and 30.3% respectively. The cohesion of reinforced sand increases with an increase in dry density. The internal friction angle firstly increases, and then stabilizes with an increase in dry density. In general, the specimens with high compactness exhibit strong shear strength when the polymer and fiber contents remain the same.

The shear strengths of reinforced sand under 100, 200, 300 and 400 kPa of vertical pressure were calculated and the changing curve is shown in Figure 11b. Although the internal friction angle of the reinforced sand varies little with an increase in dry density, the shear strength still shows a significant increase. As seen, the shear strengths at different vertical pressures of 1.60 g/cm^3^ dry density reinforced sand are 102.23, 156.12, 210.02 and 263.92 kPa which are 1.50, 1.44, 1.42 and 1.40 times compared to the specimens at 1.40 g/cm^3^ dry density.

### 4.4. Tensile Test

#### 4.4.1. Effect of Polymer Content

**Figure 12:**
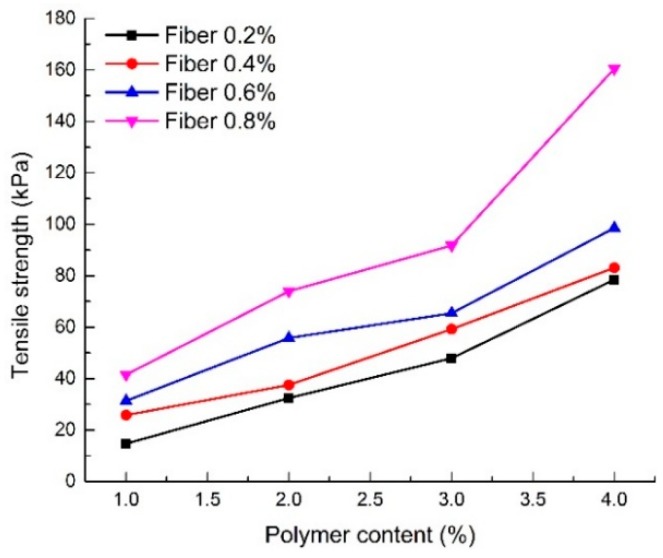
Tensile strength of reinforced sand with different polymer content.

Figure 12 shows the relationship between the tensile strength of the reinforced sand and different polymer contents (S1–S16). For 0.4% fiber content, the tensile strength of reinforced sand increased from 25.75 to 83.1 kPa when the polymer content increased from 1% to 4%. Moreover, different variation characteristics are found in specimens at 0.8% fiber content compared with specimens at the other three fiber contents. The increment rate increased with increasing polymer content. High polymer content was the main cause of this appearance, which means that the polymer can be more evenly and widely distributed in specimens with high polymer content.

The reinforced sand gradually changes from brittle to plastic as polymer content increases. Polymers can efficiently impede the production and development of tensile cracks, which led the specimens from brittle failure to plastic failure. The beneficial effects of polymer reinforcement on tensile strength can be attributed to the increase in the bonding force between sand particles and the interfacial force between fibers and sand particles.

#### 4.4.2. Effect of Fiber Content

**Figure 13:**
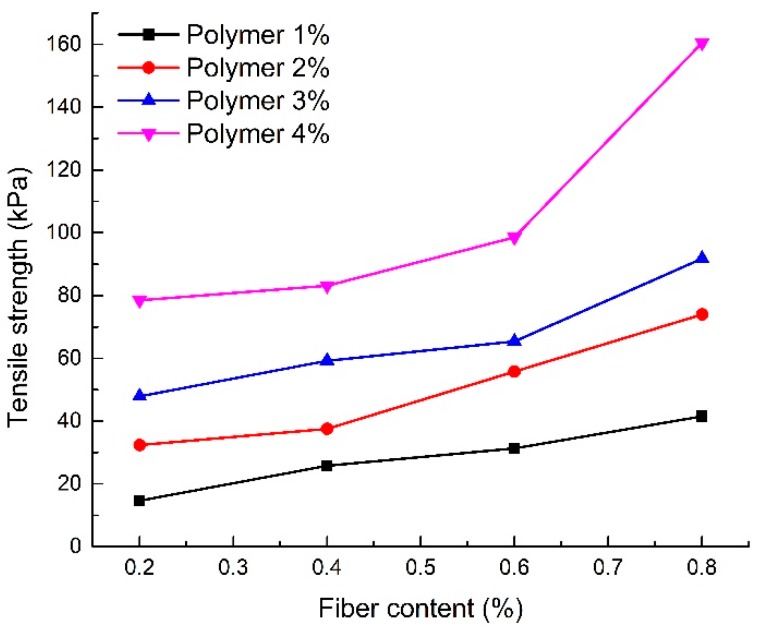
Tensile strength of reinforced sand with different fiber content.

Figure 13 shows the relationship between the tensile strength of reinforced sand and different fiber contents (S1–S16). The tensile strength increased monotonically with fiber content. For the specimen with 2% polymer content, the tensile strength increased from 32.38 to 73.96 kPa when the fiber content increased from 0.2% to 0.8%. This increase in the tensile strength demonstrates the effectiveness of fiber glass in sand reinforcement.

Figure 13 also shows that the beneficial effects of fiber reinforcement on tensile strength are affected by polymer content. The tensile strength tends to increase faster with an increase in fiber content when the polymer content is high. When the polymer content is low, the increasing trend of tensile strength with fiber content is also low. Tang et al. [23] investigated the interfacial shear strength of fiber reinforced sand and found that the fiber reinforcement effect was determined by the bonding strength and friction between fibers and sand particles. The cohesion provided by the polymer enhanced the bonding strength and friction. It is considered that compaction and cementation enable fibers a full play in reinforcement. This explains the discrepancy in the effects of fibers on the tensile strength when the polymer content is different.

#### 4.4.3. Effect of Dry Density

In order to better understand the effect of dry density on the tensile strength of reinforced sand, an additional eight groups of samples were prepared. The relevant parameters and the tensile test results of additional specimens are shown in Table 5.

**Figure 14:**
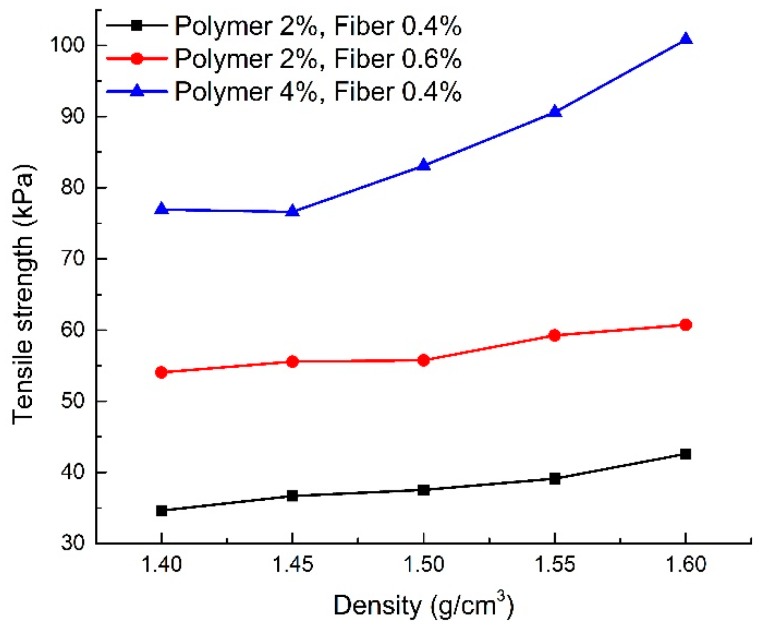
Tensile strength of reinforced sand with different dry density.

Figure 14 shows the tensile strength of specimens at different dry densities (S6, S17–S20 and S21–S28). Three proportions shown in Table 3, Table 4 and Table 5 were adopted to investigate the effect of dry density. As shown in Figure 14, the tensile strength of sand increases with an increase in dry density. For instance, the tensile strength increased by approximately 23.0% when the dry density increased from 1.40 to 1.60 g/cm^3^ with 2% polymer content and 0.4% fiber content. This is mainly due to the decrease in the porosity of the sample with an increase in dry density. The connection point and contact area of each sand particle in the reinforced sand increased; therefore, the capacity for tensile stress is strengthened. In addition, an increase in dry density also increases the contact area between the surface of the fiber glass and the sand particles to improve the interfacial force. Tang et al. [23] found that the interfacial force between the fiber and soil particles increased by 2.2 times when the dry density increased from 1.4 to 1.6 g/cm^3^ by the tensile test of single fiber. Therefore, the anti-slip ability and the tensile strength of fiber in reinforced sand can increase with an increase in dry density.

Comparing the three curves, it is found that an increase in polymer content can enhance the influence of dry density on the tensile strength of sand. The tensile strength increased by approximately 23.0% when the dry density increased from 1.40 to 1.60 g/cm^3^ with 2% polymer content and 0.4% fiber content. When the fiber content is constant and the polymer content is increased by 2%, the tensile strength increased by approximately 31.0% with the dry density increasing from 1.40 to 1.60 g/cm^3^. The increase in fiber content can also enhance the influence of dry density on the tensile strength of sand. In comparison, this effect is poor.

## 5. Discussion

The results show that the compaction and a certain amount of a water-based organic polymer and fiber glass can significantly improve the strength of sand. However, it is not a simple linear relationship between the compaction and the amount of water-based organic polymer and fiber glass to enhance the effect of sand strength. These are all associated with the mechanism of the water-based organic polymer and fiber glass reinforced sand. At present, there are few studies about the mechanism of water-based organic polymer and fiber glass reinforced sand. The reinforcement mechanism is discussed in the following three sections in this study.

### 5.1. Effect of Water-Based Organic Polymer

The water-based organic polymer used in this study is mainly composed of polyurethane resin. Its chemical formula is shown in Formula (4). A chemical reaction occurred when it came into contact with water. The specific reaction process is shown in Formulas (5) and (6).

(4) $$O=C=N\;{\left[{\;R}_{1}-\mathrm{NH}-\mathrm{CO}-R_{2}-O-\mathrm{CO}-\mathrm{NH}\;\right]}_{n}{\;R}_{1}-N=C=O,$$

(5) $$\;O=C=N-R-N=C=O+2H_{2}O\to \mathrm{HO}-\mathrm{CO}-\mathrm{NH}-R-\mathrm{NH}-\mathrm{CO}-\mathrm{OH}\to H_{2}N-R-{\mathrm{NH}}_{2}+2{\mathrm{CO}}_{2}$$

(6) $$\left(n+1\right)H_{2}N-R-{\mathrm{NH}}_{2}+nO=C=N-R-N=C=O\to H_{2}N\;{\left[\;R-\mathrm{NH}-\mathrm{CO}-\mathrm{NH}\;\right]}_{2n}\;R-{\mathrm{NH}}_{2},$$

**Figure 15:**
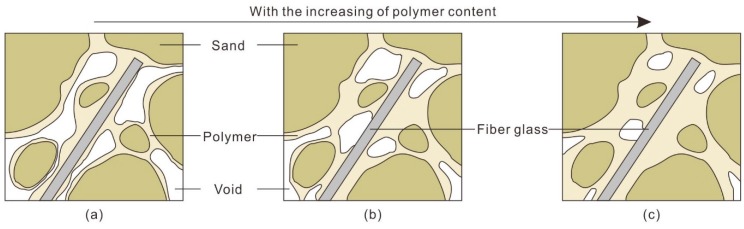
Schematic drawing of the microstructure of reinforced sand at different polymer contents. (**a**) Low polymer content; (**b**) Medium polymer content; (**c**) High polymer content.

The reinforcement mechanism of the water-based organic polymer can be categorized as enwrapping, filling and connecting. At preparation, the polymer solution was fully mixed with sand, and it formed a membrane that enwraps sand particles and fibers. Meanwhile, the polymer solution can fill the voids and connect sand particles and fibers. A space network membrane structure forms in the specimen. With an increase in polymer content, the membrane structure gradually improves. Figure 15 shows the structural change in reinforced sand with an increasing polymer content. As shown in Figure 15a, the number and volume of voids in the reinforced sand is large when the polymer content is low. With an increase in polymer content, the filling and connecting effect of polymer is enhanced which results in a decrease of voids in number and volume (Figure 15b,c). Furthermore, the integrity of the sand is increased.

In addition, the failure form of sand gradually changes from brittle to plastic with an increasing polymer content. The beneficial effects of polymer reinforcement on strength can be attributed to the increase in bonding force between sand and the interfacial force between fibers and sand. With an increase in polymer content, fibers are more closely bonded with the sand, and the interfacial forces are increased by increasing the contact area between them. Moreover, the same effect is also seen on the sand particles, which is presented as welding the separate sand particles together by cementation. This is main reason that the strength of reinforced sand is improved.

### 5.2. Effect of Fiber Glass

**Figure 16:**
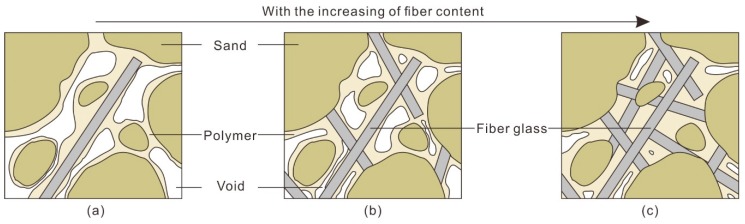
Schematic drawing of the microstructure of reinforced sand at different fiber contents. (**a**) Low fiber content; (**b**) Medium fiber content; (**c**) High fiber content.

The reinforcing mechanism of fiber glass mainly includes the reinforcing effect of a single fiber and the reinforcing effect of the fiber net. Figure 16 shows the structural change of reinforced sand with an increasing fiber content. When the content of fiber glass is rather little (Figure 16a), the spacing of the fibers is large. The fiber glass is isolated in the sandy soil and is surrounded by sand particles. Because the elastic modulus of the fiber glass is much higher than that of the sand, when the force is stressed, the inconsistency of the deformation will inevitably lead to the interposition of the sand particles and the fiber glass. This causes the fiber glass to be pulled to produce an interfacial force, which can limit the relative sliding of the fiber glass so that the external load is shared. In addition, when the fiber is drawn, it will restrain the sand particles and limit the deformation of the sand, which can also improve the mechanical properties of the sand. When a large number of fibers are randomly distributed in the sand (Figure 16b,c), they are instead woven into a net. It is necessary to pull the other fibers together to form a three-dimensional force network when one of the fibers is pulled. This ensures that the load is distributed to a wider area and further improves the tensile strength of the fiber.

Different fiber contents can influence the effect of fiber glass on the strength of sand. When the fiber content is low, the effective fiber glass net cannot be formed due to the large fiber spacing. The contribution of fiber glass to sand strength mainly comes from the reinforcing effect of a single fiber. With the increase in fiber content, the fiber glass begins to inter weave into a net. The contribution of fiber glass to sand strength is not only the reinforcing effect of a single fiber, but also the reinforcing effect of the fiber net, which leads to an increase in sand strength in the high fiber content interval.

### 5.3. Effect of Dry Density

**Figure 17:**
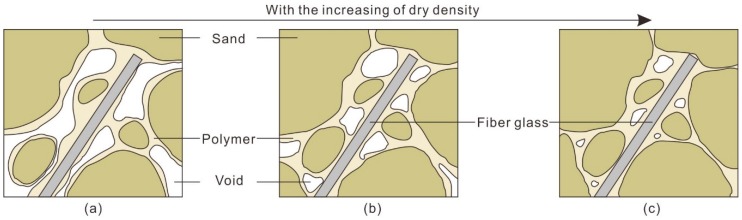
Schematic drawing of microstructure of reinforced sand at different dry density. (**a**) Low dry density; (**b**) Medium dry density; (**c**) High dry density.

The influence of dry density on the strength of reinforced sand is mainly reflected in the change of the contact relation between the polymer, fiber glass and sand particles. Figure 17 shows the structural change of reinforced sand with increasing dry density. For sand with a small dry density, the voids in the sand are relatively large, resulting in an insufficient filling effect of the polymer. In this case, the membrane caused by the water-based organic polymer tends to enwrap sand particles and fibers instead of connecting. As the dry density increases, the voids between the sand particles and the fiber glass decrease. The connecting effect of the polymer is gradually strengthened so that the interfacial force between the sand particles and the fiber glass increases. This has a positive effect on the increase in sand strength. In addition, it requires a high pressure to prepare the specimen with a high dry density. Sand particles can damage the surface of the fiber glass or cause the plastic deformation of the fiber glass, which increases the roughness of the surface of the fiber and improves the mechanical effect of the interface.

### 5.4. Results and Analysis of Scanning Electron Microscopy (SEM)

**Figure 18:**
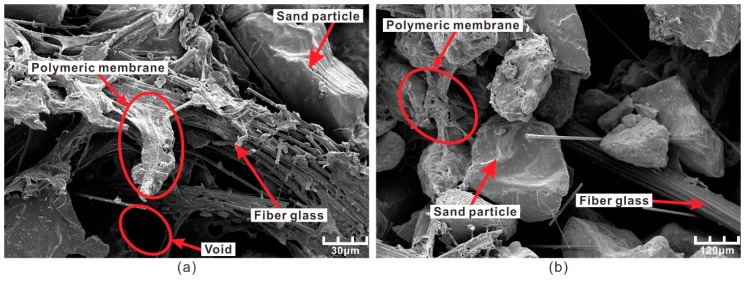
Scanning electron microscopy (SEM) micrograph of fiber-sand mixture: (**a**) Polymeric membrane; (**b**) Anchorage effects.

After strength testing, the samples were subjected to scanning electron microscopy (SEM) to obtain the microscopic state of the reinforced sand. Figure 18 shows the SEM micrographs of reinforced sand at different magnifications. Figure 18a shows that a membrane, formed by the polymer, widely exists inside the reinforced sand. It enwrapped and connected the sand particles and fibers. In addition, the polymeric membrane increased the bonding and interlocking forces between sand particles which made the loose sand and fibers more integrated. With an increase in polymer content, the membrane can enwrap and connect sand particles more tightly which causes the strength to increase.

Figure 18b shows that the fiber glass is interspersed into the voids of the sand particles. Due to the polymeric membrane, the fiber glass is anchored in the void which makes the fiber difficult to pull out. As a result, fibers were able to bear some load caused by external stress and distribute the stress to a broader area. With an increase in fiber content, the connections between the fiber and sand increased the integrity of the sand. With increasing polymer content, the fiber is firmly anchored at both ends by the bonding force which creates stronger reinforcement effects.

## 6. Conclusions

Three strength tests (unconfined compression test, direct shear test and tensile test) and scanning electron microscopy were applied to identify the reinforcement effects of a type of water-based organic polymer and fiber glass on sand. The effects of fiber content, polymer content and dry density on sand strength were investigated. Based on the results obtained, the following conclusions can be drawn:

(1) The unconfined compression strength of sand can be effectively improved by the water-based organic polymer and fiber glass. When the dry density remains the same, the strength increases with increasing polymer and fiber contents. When the polymer and the fiber contents remain the same, the strength increases with an increase in dry density.

(2) The cohesion of sand can be improved effectively by adding the water-based organic polymer and fiber glass to sand. With an increase in polymer and fiber content, the cohesion increases. The trend is also the same with increasing dry density. However, the situation is different for internal friction angles. With an increase in polymer content and dry density, the internal friction angle of sandy soil also increases. With an increase in fiber content, the internal friction angle of sandy soil remains relatively stable.

(3) The addition of the polymer and fiber glass into sand and the subsequent compaction of the sand significantly improves its tensile strength. The tensile strength increased monotonically with an increase in polymer content, fiber content and dry density within the test range. When there is a large content of polymer and fiber, the growth trend of the tensile strength of sand is pronounced.

(4) The reinforcement mechanism of the polymer is generated by a polymeric membrane structure. The effects of the membrane are categorized as enwrapping, filling and connecting. The reinforcing mechanism of fiber glass mainly includes the reinforcing effect of a single fiber and the reinforcing effect of the fiber net. Both of these can increase the interfacial force and prevent sand particles from rearranging under load. The influence of dry density on the strength of reinforced sand is mainly reflected in the change in the contact relation between the polymer, fiber glass and sand particles.

## Figures and Tables

**Table 1:** The detailed physical parameters of sand.

*G*s	*ρ*_max_ (g/cm^3^)	*ρ*_min_ (g/cm^3^)	*e*_max_	*e*_min_	*D*_50_ (mm)	*C*_c_	*C*_u_
2.64	1.66	1.34	0.970	0.590	0.30	1.05	2.27

**Table 2:** The basic physical and mechanical parameters of the fiber glass.

Density (g/cm^3^)	Diameter (mm)	Elongation at Break (%)	Tensile Strength (MPa)	Elastic Modulus (GPa)	Melting Point (°C)
2.49	0.08	2~3	3100~4650	73~86	680

**Table 3:** The relevant parameters of specimens.

Number	Dry Density (g/cm^3^)/Standard Deviations (g/cm^3^)	Fiber Content (%)	Polymer Content (%)	Number	Dry Density (g/cm^3^)/Standard Deviations (g/cm^3^)	Fiber Content (%)	Polymer Content (%)
S1	1.50/0.01	0.2	1	S11	1.50/0.03	0.6	3
S2	1.50/0.02	0.2	2	S12	1.50/0.03	0.6	4
S3	1.50/0.02	0.2	3	S13	1.50/0.01	0.8	1
S4	1.50/0.00	0.2	4	S14	1.50/0.03	0.8	2
S5	1.50/0.04	0.4	1	S15	1.50/0.02	0.8	3
S6	1.50/0.00	0.4	2	S16	1.50/0.01	0.8	4
S7	1.50/0.04	0.4	3	S17	1.40/0.03	0.4	2
S8	1.50/0.01	0.4	4	S18	1.45/0.02	0.4	2
S9	1.50/0.02	0.6	1	S19	1.55/0.05	0.4	2
S10	1.50/0.03	0.6	2	S20	1.60/0.01	0.4	2

**Table 4:** The results of the strength tests.

Number	Unconfined Compression Strength (kPa)/Standard Deviations (kPa)	Residual Strength (kPa)/Standard Deviations (kPa)	Elasticity Modulus (MPa)/Standard Deviations (MPa)	Cohesion (kPa)/Standard Deviations (kPa)	Internal Friction Angle (°)/Standard Deviations (°)	Tensile Strength (kPa)/Standard Deviations (kPa)
S1	118.34/2.32	36.19/0.73	2.09/0.05	11.26/0.10	27.72/0.51	14.65/0.02
S2	200.60/4.15	83.79/1.02	4.01/0.00	29.81/0.86	28.85/0.78	32.38/0.38
S3	231.97/5.33	160.65/3.86	5.21/0.13	38.94/0.88	30.02/0.67	47.88/0.53
S4	274.85/6.69	233.61/4.47	6.79/0.01	63.56/1.15	30.90/0.66	78.46/0.59
S5	118.57/3.24	42.55/1.14	2.13/0.02	15.19/0.09	27.35/0.24	25.75/0.62
S6	203.06/2.85	91.46/0.95	4.06/0.10	34.33/0.49	28.04/0.05	37.53/0.54
S7	239.15/4.87	169.96/5.02	5.71/0.14	43.46/0.93	29.40/0.59	59.20/1.63
S8	304.61/7.90	280.69/8.19	7.52/0.14	84.23/0.14	29.81/0.56	83.10/2.19
S9	140.03/3.00	53.02/0.10	2.19/0.01	20.48/0.00	26.85/0.50	31.30/0.08
S10	222.11/1.91	105.56/3.16	4.22/0.10	36.73/0.86	27.82/0.63	55.75/0.03
S11	267.97/1.56	179.15/4.75	6.00/0.03	45.58/1.04	28.11/0.73	65.40/0.03
S12	333.92/9.12	317.07/1.92	9.18/0.24	96.92/2.66	28.36/0.73	98.54/2.65
S13	147.80/1.79	64.14/0.98	4.05/0.01	27.88/0.33	26.05/0.40	41.52/0.09
S14	246.05/2.69	125.60/2.27	5.53/0.09	39.61/0.84	26.93/0.01	73.96/0.35
S15	282.36/1.23	190.27/0.30	7.24/0.00	59.61/0.22	27.64/0.09	91.76/0.86
S16	388.60/0.44	384.93/2.73	9.56/0.22	103.65/1.08	28.15/0.09	160.52/1.34
S17	112.27/2.04	86.29/1.04	2.81/0.07	28.45/0.14	20.96/0.09	34.63/0.94
S18	133.77/2.95	94.40/2.01	3.34/0.04	31.44/0.74	25.04/0.09	36.67/0.52
S19	203.06/0.67	91.46/1.42	5.08/0.09	41.32/0.32	26.62/0.41	39.12/0.63
S20	254.55/6.17	125.53/0.89	6.36/0.12	48.33/0.35	27.31/0.73	42.60/1.02

**Table 5:** The relevant parameters and the tensile test results of additional specimens.

Number	Dry Density (g/cm^3^)/Standard Deviations (g/cm^3^)	Fiber Content (%)	Polymer Content (%)	Tensile Strength (kPa)/Standard Deviations (kPa)
S21	1.40/0.02	2	0.6	54.05/1.57
S22	1.45/0.03	2	0.6	55.56/1.31
S23	1.55/0.03	2	0.6	59.26/1.00
S24	1.60/0.04	2	0.6	60.72/1.06
S25	1.40/0.03	4	0.4	76.94/0.53
S26	1.45/0.02	4	0.4	76.60/0.39
S27	1.55/0.02	4	0.4	90.59/1.44
S28	1.60/0.01	4	0.4	100.79/1.73
